# Initial data release and announcement of the 10,000 Fish Genomes Project (Fish10K)

**DOI:** 10.1093/gigascience/giaa080

**Published:** 2020-08-18

**Authors:** Guangyi Fan, Yue Song, Liandong Yang, Xiaoyun Huang, Suyu Zhang, Mengqi Zhang, Xianwei Yang, Yue Chang, He Zhang, Yongxin Li, Shanshan Liu, Lili Yu, Jeffery Chu, Inge Seim, Chenguang Feng, Thomas J Near, Rod A Wing, Wen Wang, Kun Wang, Jing Wang, Xun Xu, Huanming Yang, Xin Liu, Nansheng Chen, Shunping He

**Affiliations:** BGI-Qingdao, 2 Hengyunshan Road, West Coast New Area, 266426, Qingdao, China; BGI-Shenzhen, Building 11, Beishan Industrial Zone, Yantian District, Shenzhen, 518083, China; State Key Laboratory of Agricultural Genomics, BGI-Shenzhen, Building 11, Beishan Industrial Zone, Yantian District, Shenzhen, 518083, China; BGI-Qingdao, 2 Hengyunshan Road, West Coast New Area, 266426, Qingdao, China; Key Laboratory of Aquatic Biodiversity and Conservation, Institute of Hydrobiology, Chinese Academy of Sciences, No. 7 Donghu South Road, Wuchang District, Wuhan, Hubei Province, China; BGI-Qingdao, 2 Hengyunshan Road, West Coast New Area, 266426, Qingdao, China; BGI-Qingdao, 2 Hengyunshan Road, West Coast New Area, 266426, Qingdao, China; BGI-Qingdao, 2 Hengyunshan Road, West Coast New Area, 266426, Qingdao, China; BGI-Qingdao, 2 Hengyunshan Road, West Coast New Area, 266426, Qingdao, China; BGI-Qingdao, 2 Hengyunshan Road, West Coast New Area, 266426, Qingdao, China; BGI-Qingdao, 2 Hengyunshan Road, West Coast New Area, 266426, Qingdao, China; BGI-Shenzhen, Building 11, Beishan Industrial Zone, Yantian District, Shenzhen, 518083, China; Center for Ecological and Environmental Sciences, Northwestern Polytechnical University, 1 Dongxiang Road, Chang'an District, Xi'an Shaanxi,710129, China; BGI-Qingdao, 2 Hengyunshan Road, West Coast New Area, 266426, Qingdao, China; BGI-Qingdao, 2 Hengyunshan Road, West Coast New Area, 266426, Qingdao, China; Frasergen, Donghu High-Tech Development Zone, Donghu High-Tech Development Zone, Wuhan, Hubei Province, China; Integrative Biology Laboratory, College of Life Sciences, Nanjing Normal University, No.1 Wenyuan Road Qixia District, Nanjing, 210023, China; Comparative and Endocrine Biology Laboratory, Translational Research Institute-Institute of Health and Biomedical Innovation, School of Biomedical Sciences, Queensland University of Technology, Brisbane 4102, Queensland, Australia; Center for Ecological and Environmental Sciences, Northwestern Polytechnical University, 1 Dongxiang Road, Chang'an District, Xi'an Shaanxi,710129, China; Department of Ecology & Evolutionary Biology, Yale University, New Haven, CT 06511, USA; Biological and Environmental Sciences & Engineering Division, King Abdullah University of Science and Technology, Thuwal 23955-6900, Kingdom of Saudi Arabia; Center for Ecological and Environmental Sciences, Northwestern Polytechnical University, 1 Dongxiang Road, Chang'an District, Xi'an Shaanxi,710129, China; Center for Ecological and Environmental Sciences, Northwestern Polytechnical University, 1 Dongxiang Road, Chang'an District, Xi'an Shaanxi,710129, China; Key Laboratory of Marine Ecology and Environmental Sciences, Institute of Oceanology, Chinese Academy of Sciences, 7 Nanhai Road, Qingdao, Shandong 266071, China; Marine Ecology and Environmental Science Laboratory, Pilot National Laboratory for Marine Science and Technology, 1 Wenhai Road,Aoshanwei,Jimo, Qingdao,Shandong, 266237, China; Center for Ocean Mega-Science, Chinese Academy of Sciences, No. 7, Nanhai Road, Qingdao City, 266400, China; BGI-Shenzhen, Building 11, Beishan Industrial Zone, Yantian District, Shenzhen, 518083, China; BGI-Qingdao, 2 Hengyunshan Road, West Coast New Area, 266426, Qingdao, China; BGI-Shenzhen, Building 11, Beishan Industrial Zone, Yantian District, Shenzhen, 518083, China; BGI-Qingdao, 2 Hengyunshan Road, West Coast New Area, 266426, Qingdao, China; BGI-Shenzhen, Building 11, Beishan Industrial Zone, Yantian District, Shenzhen, 518083, China; State Key Laboratory of Agricultural Genomics, BGI-Shenzhen, Building 11, Beishan Industrial Zone, Yantian District, Shenzhen, 518083, China; Key Laboratory of Marine Ecology and Environmental Sciences, Institute of Oceanology, Chinese Academy of Sciences, 7 Nanhai Road, Qingdao, Shandong 266071, China; Marine Ecology and Environmental Science Laboratory, Pilot National Laboratory for Marine Science and Technology, 1 Wenhai Road,Aoshanwei,Jimo, Qingdao,Shandong, 266237, China; Center for Ocean Mega-Science, Chinese Academy of Sciences, No. 7, Nanhai Road, Qingdao City, 266400, China; Department of Molecular Biology and Biochemistry, Simon Fraser University, Burnaby, BC, V5A 1S6, Canada; Key Laboratory of Aquatic Biodiversity and Conservation, Institute of Hydrobiology, Chinese Academy of Sciences, No. 7 Donghu South Road, Wuchang District, Wuhan, Hubei Province, China

**Keywords:** Fish10K, fish, genome sequencing, phylogenetics, evolution, stLFR

## Abstract

**Background:**

With more than 30,000 species, fish—including bony, jawless, and cartilaginous fish—are the largest vertebrate group, and include some of the earliest vertebrates. Despite their critical roles in many ecosystems and human society, fish genomics lags behind work on birds and mammals. This severely limits our understanding of evolution and hinders progress on the conservation and sustainable utilization of fish.

**Results:**

Here, we announce the Fish10K project, a portion of the Earth BioGenome Project aiming to sequence 10,000 representative fish genomes in a systematic fashion within 10 years, and we officially welcome collaborators to join this effort. As a step towards this goal, we herein describe a feasible workflow for the procurement and storage of biospecimens, as well as sequencing and assembly strategies.

**Conclusions:**

To illustrate, we present the genomes of 10 fish species from a cohort of 93 species chosen for technology development.

## Introduction

### Fish genomes sequenced to date

**Figure 1:**
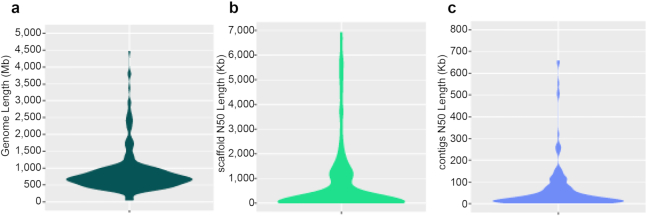
Assembly statistics of fish genomes in public databases. (**a**) Summary of genome size. (**b, c**) N50 statistics. A scaffold is a set of contigs linked together with gaps introduced in between. N50 is the median contig size of the genomic assembly. It's a metric that could be used to evaluate the quality of genome assembly.

As of the time of this writing, genome assemblies are publicly available for fewer than 1% of fish species (244 species, as sourced from NCBI when accessed on 21 April 2020; Supplementary Table 1). Their assembly lengths range from 302.36 Mb (*Diretmus argenteus*) to 4.47 Gb (*Scyliorhinus torazame*), with an average length of 872.64 Mb. The average scaffold N50 and contig N50 values are 8.82 Mb and 914.18 Kb, respectively, while the median scaffold N50 and contig N50 are 613.59 Kb and 20.82 Kb, respectively. There are 112 species with a scaffold N50 of more than 1 Mb, of which 43 have a contig N50 above 1 Mb (Fig. 1). These genomes have fueled a number of studies on the phylogeny and evolution of fish (e.g., the African coelacanth), evolutionary processes of specific fish subgroups (e.g., elephant shark genome illustrating the phylogenetic relationship of Chondrichthyes as a sister group to bony vertebrates), genetic mechanisms of adaptation to different environments (e.g., the deep-sea Mariana Trench snailfish and cave-dwelling fish), and specific biological processes (e.g., the evolutionary process of ZW sex chromosomes). Nevertheless, the current fish genome sequencing results are only a drop in the ocean, and numerous critical research questions remain to be resolved. A non-exhaustive list includes gaining comprehensive and clear understandings of fish phylogeny, genome size diversity and chromosome evolution, diverse environmental adaptations, morphology evolution, respiratory system, immune system, and the evolution and function of ultraconserved elements and conserved nonexonic elements.

### The era of genome consortiums

With the rapid development of DNA sequencing technology, this is the time for large-scale, collaborative genomic studies to map the vertebrate tree of life. The first such project was the Genome 10K, established in 2009, which aimed to sequence and assemble genomes of about 10,000 vertebrate species [1]. The Earth BioGenome Project aims to sequence, catalog, and characterize the genomes of all of Earth's eukaryotic biodiversity [2]. The Vertebrate Genomes Project (VGP) was launched in 2017 to generate chromosome-level, haplotype-phased genome assemblies of all vertebrate species [3]. The Bird 10,000 Genomes Project (B10K) was initiated [4] after a successful phylogenomic study on 45 avian genomes in 2014. The B10K projects aims to sequence and assemble all known bird species in 3 phases. Despite current challenges in funding, sampling, sequencing, assembly, and data analysis, these projects have already made substantial progress. For fish, which make up more than half of all vertebrate species, no exclusive fish genome projects have been initiated at a similar scale. To our knowledge, the only large-scale genomic study was Fish-T1K (Transcriptomes of 1,000 Fishes), which aimed to sequence the transcriptomes (RNA sequence) of ray-finned fish [5]. However, the insights gained from transcriptome data alone are relatively limited. Accelerating fish genomics by large-scale genome sequencing efforts would undoubtedly boost research into fish biodiversity, speciation, and adaptation, as well as aiding the conservation and sustainable utilization of fish.

### The Fish10K Genome Project

We here announce the Fish10K Genome Project, a sub-project of the Earth BioGenome Project aiming to sample, sequence, assemble, and analyze genomes of 10,000 fish species. We are proposing an effective and integrated workflow in which major challenges are addressed and in which high-quality reference genomes are constructed (chromosome level in Phases I and II and a scaffold-level assembly with a scaffold N50 larger than 1Mb in Phase III). Through developing and applying effective analysis methods, we will be able to address critical evolutionary and biological research questions related to fish.

In order to prove the efficiency of our workflow and the feasibility of this large-scale genome project, 10 species from 93 collected samples are used to validate our new sequencing technology, and these genomes have been released as part of a pilot project.

## Materials and Methods

### Feasibility test and the release of 10 fish genomes

**Figure 2:**
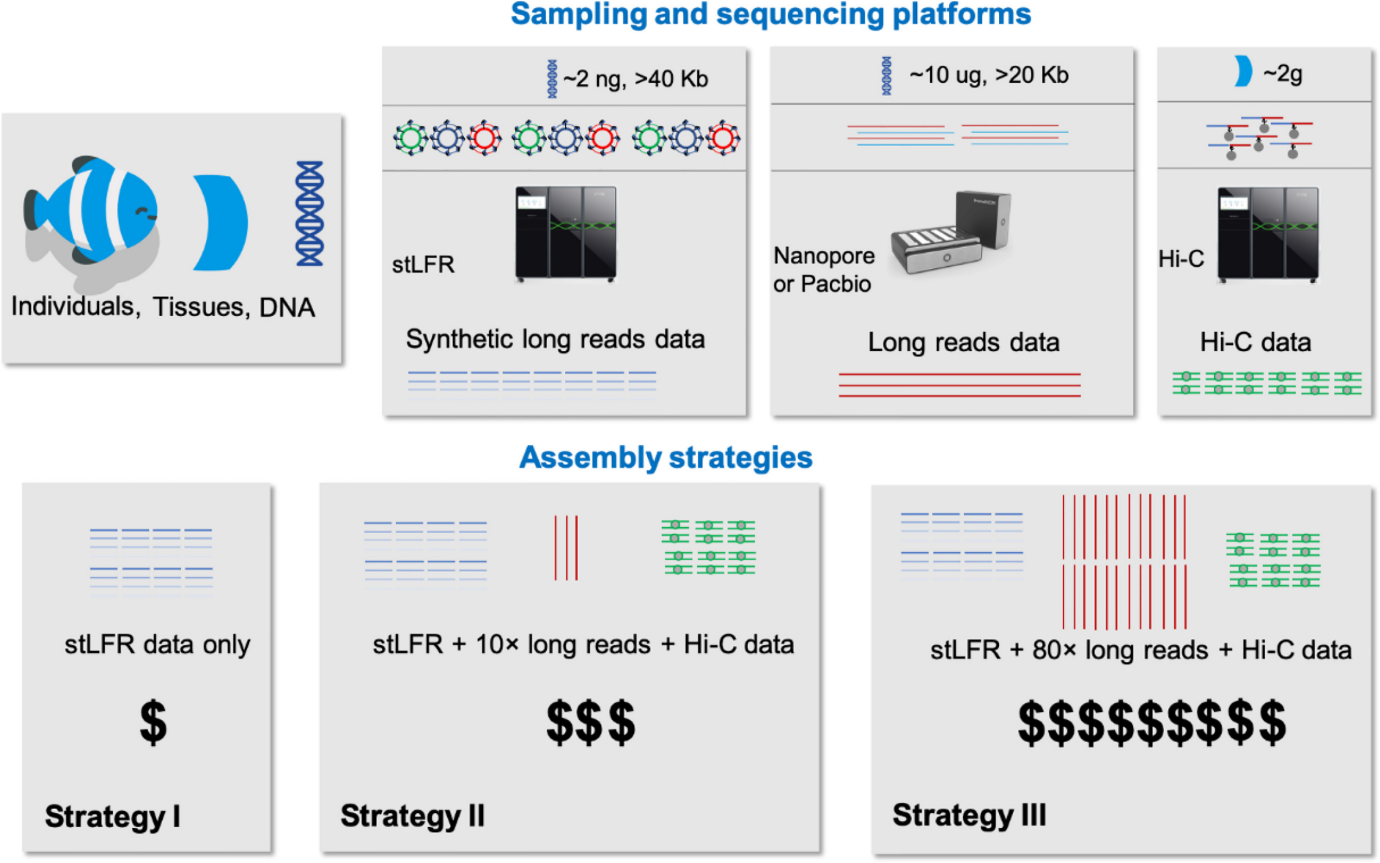
The sequencing and assembly strategies. In the preferred strategy (Strategy II), high-quality DNA fragments (≥40Kb) are used to construct a stLFR library, which is sequenced using the DNBSEQ platform. Low–sequencing depth long reads are only used to improve the continuity of highly complex regions (increase the contig N50). In the alternative Strategy I, high-depth long reads are used to construct contigs, while low-depth stLFR reads are used to polish the contig and link the scaffolds. Hi-C data are used to generate a chromosome-level assembly.

In order to establish cost-effective strategies and assess the feasibility of a large-scale genome project, we initiated a pilot study in June 2017. Over the next 2 years, we went on 4 expeditions across lakes, rivers, and coastal waters of China, collecting 324 fish species. After careful documentation of sample information and species identification, the tissues of 93 species were selected for DNA extraction, and 10 of these species were used for sequencing. We used single-tube long fragment reads technology (stLFR) [6] and the DNBSEQ platform to sequence the species, generating long-read (Nanopore or PacBio) and Hi-C data for a subset of the species. In this way, we were able to test the feasibility of 3 different sequencing and assembly strategies (Fig. 2): stLFR data alone (synthetic long reads generated using a second-generation sequencing platform; Strategy I); stLFR data combined with low-depth, long reads (~10 × raw Nanopore data to fill in the gaps; Strategy II); and high-depth, long reads (~80 × raw Nanopore data) combined with second-generation short reads (either short insert size libraries or stLFR; Strategy III; Table 1 and Table 2). To date, we have assembled the genomes of the first 10 species. For the 10 assembled fish genomes, the average contig N50 and the average scaffold N50 are 2.83 Mb and 7.59 Mb, respectively. The average benchmarking universal single-copy orthologs (BUSCO) completeness estimate is 96%. A comparison of assembly statistics revealed that assemblies generated with Strategy II and Strategy III were more continuous. To illustrate our effort, we are releasing the genomes of 10 representative bony fish genomes covering the 3 assembly strategies. The contig N50s of 7 of these genomes are more than 1 Mb and a minimum of 93% of BUSCO genes were found, indicating the genome assemblies are of high quality. There were 3 genomes assembled at the chromosome level, with more than 92% of scaffolds anchored using Hi-C data.

**Table 1:** Assembly statistics of the 10 released genome assemblies

Strategy	Species	Common name	Estimated genome size, Mb	Assembly size, Mb	Scaffold N50, bp	Contig N50, bp	BUSCO, %	Anchored, %
**I**	*Diodon holocanthus*	Longspined porcupinefish	722.9	643.4	6,098,089	2,149,931	95.7	**–**
	*Heterotis niloticus^a^*	African bonytongue	743.4	669.7	9,615,753	2,307,881	97.6	96.8
	*Oxyeleotris marmorata*	Marble goby	589.7	502.6	13,190,768	1,270,297	92.9	**–**
	*Datnioides undecimradiatus*	Mekong tiger perch	623.1	595.7	9,741,635	2,175,996	97.2	**–**
	*Chaetodon trifasciatus*	Melon butterflyfish	698.5	668.3	9,974,986	1,859,054	97.3	**–**
**II**	*Naso vlamingii*	Bignose unicornfish	961.4	861.3	5,736,754	182,642	97.8	**–**
	*Chelmon rostratus^a^*	Copperband butterflyfish	711.4	638.9	2,627,953	294,414	98.4	94.4
	*Helostoma temminckii* ^a^	Kissing gourami	729.7	635.4	913,351	95,536	96.3	91.8
**III**	*Pseudobrama simoni*	**–**	940.9	929.1	13,799,189	13,799,189	95.7	**–**
	*Rhodeus ocellatus*	Rosy bitterling	850.5	902.4	4,198,183	4,198,183	94.5	**–**

The common names were obtained from the FishBase website (https://www.fishbase.se/search.php). ^a^Chromosome-level genome assembly (Hi-C data generated).

**Table 2:** Sample collection template.

Species	Length, cm	Weight, g	Sex	Meta	Intestinal	Muscle	Liver	Time	Place	Longitude and latitude	Photo	Sampling person	Identification person	Status
*Sebastiscus marmoratus*	11.5	27	♂	√	√	√	√	20,190,421	Xiamen	N24°11′59.58″ E118°25′1.92″	√	Dr. Meng	Prof. He	Living
*Pisodonophis cancrivorus*	12	31	♂	√	√	√	√	20,190,421	Ningde	N24°11′59.58″ E118°25′1.92″	√	Dr. Meng	Prof. He	Fresh
*Odontobutis obscura*	13.3	35	♀	√	√	√	√	20,190,421	Hangzhou	N24°11′59.58″ E118°25′1.92″	√	Dr. Meng	Prof. He	Frozen

### The Fish10K Genome Project: from 100 to 10,000

**Figure 3:**
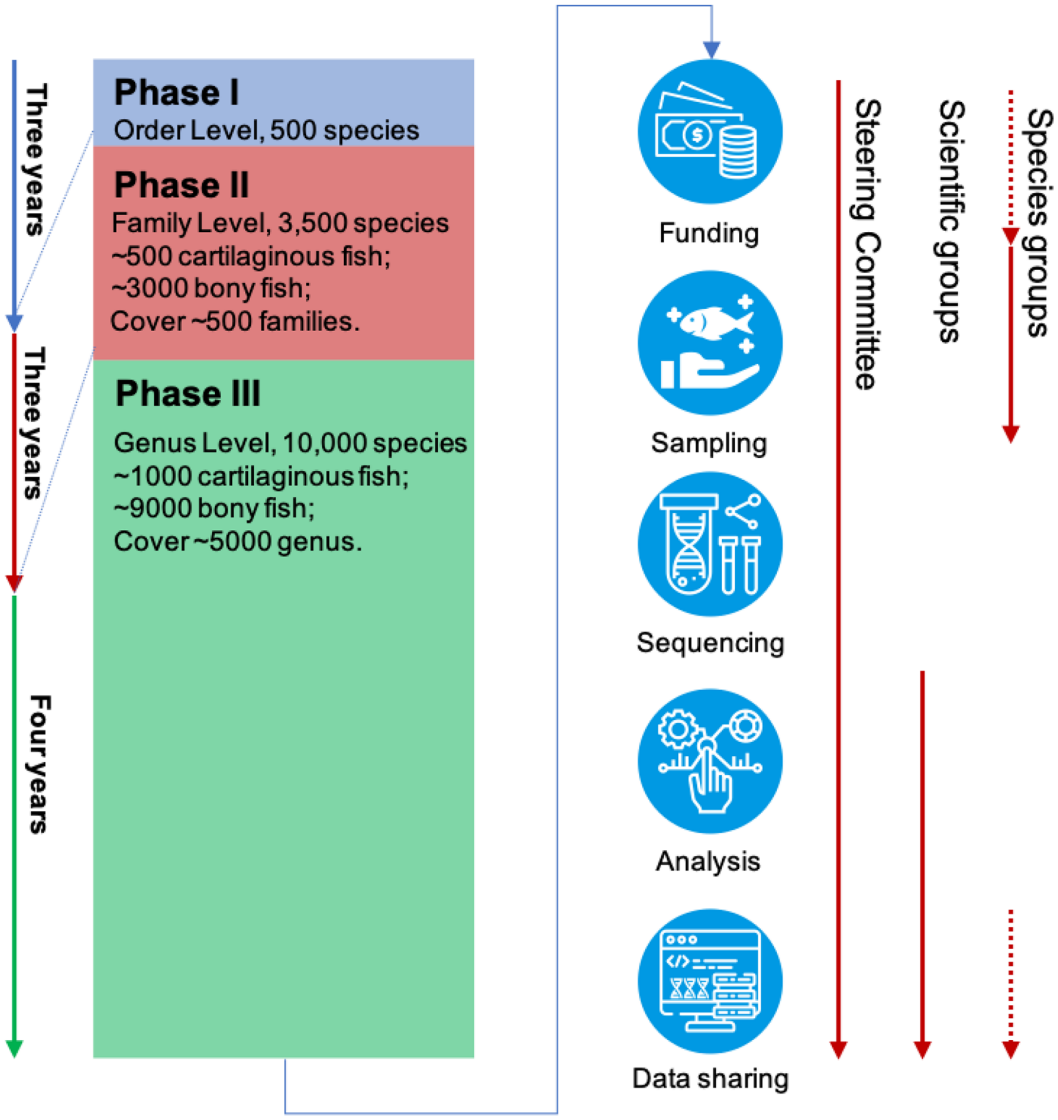
The roadmap and organization of Fish10K. Fish10K is divided into 3 phases, based on the evolutionary relationship of fish, and 3 working groups (steering committee, scientific groups, and species groups).

**Figure 4:**
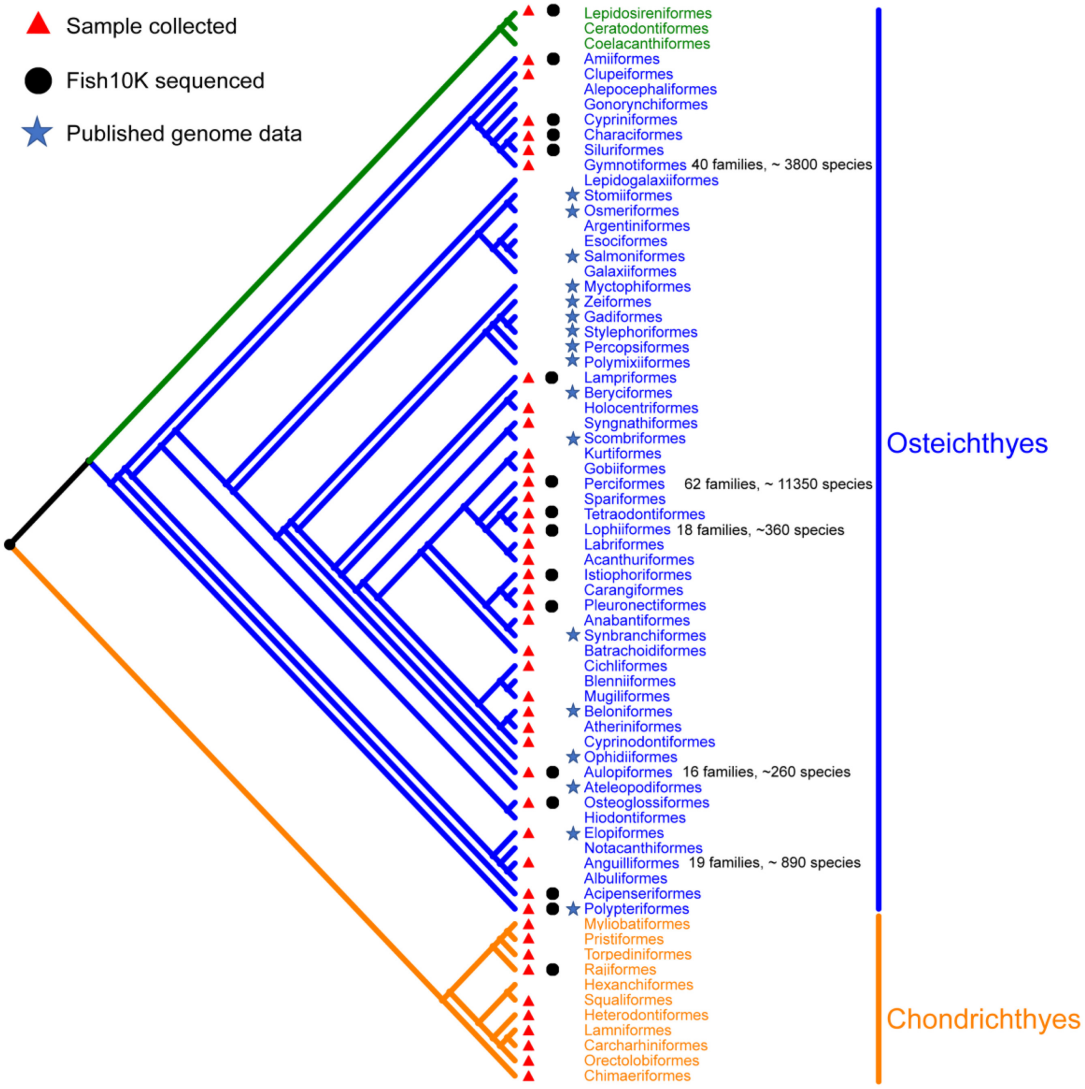
Phylogenetics tree of fish. Jawed vertebrates (gnathostomes) are divided into 2 major groups: cartilaginous fish (Chondrichthyes; in orange) and bony vertebrates (Osteichthyes; in blue and green). Bony fish are grouped into 2 subgroups (Sarcopterygii; green) and (Actinopterygii; blue). The number of families and species in the 5 largest orders are labeled. The remaining 10 orders of bony fish (Caproiformes, Callionymiformes, Gobiesociformes, Icosteiformes, Lepisosteiformes, Moroniformes, Scombrolabraciformes, Scorpaeniformes, Trachichthyiformes, and Trachiniformes) and 2 orders of cartilaginous fish (Rhinopristiformes and Squatiniformes) are not included in the phylogenetic tree, due to their uncertain positions.

With the experience gained in the Fish10K pilot study and our published results (e.g., the genome of Mekong tiger perch [*Datnioides undecimradiatus*] provides insights into the phylogenetic position of Lobotiformes and biological conservation), we believe that the project can scale up. Thus, we are proposing a roadmap (Fig. 3) in which we will construct high-quality reference genomes for representative species in all orders (Phase I) and families (Phase II), in concert with the generation of draft genome sequences for additional related species (Phase III). An interrogation of FishBase (https://www.fishbase.se) and *Fishes of the World* [7] revealed information on 34,115 fish species from ~5,000 genera, ~529 families, and ~80 orders (Supplementary Table 2). The species were divided into 6 lineages (Elasmobranchii, Holocephali, Actinopterygii, Sarcopterygii, Cyclostomes, and Myxini), in which Elasmobranchii and Holocephali belong to Chondrichthyes (cartilaginous fish) and Actinopterygii and Sarcopterygii belong to Osteichthyes (bony fish). As mentioned above, there are reference genomes available for at least 1 species each of 56 orders, while for the rest of the orders reference genomes are required (Fig. 4). Also, there are fish orders with a large number of species (e.g., Perciformes has 62 families; Siluriformes has 40 families; and Scorpaeniformes has 39 families), suggesting that additional high-quality reference genomes are required to represent the diverse biological characteristics. Thus, in Phase I we aim to sequence 450 bony fish and 50 cartilaginous fish species, covering all 80 orders (Supplementary Table 3). In Phase II, we aim to sequence approximately 3,000 species, covering almost all ~500 fish families. In Phase III, we will sequence ~6,500 fish genomes, covering ~5,000 genera.

### Sampling, sequencing, assembly, and annotation

Sampling is a critical challenge in any large-scale genome consortium. We propose a centralized sampling mode (i.e., mirroring our 93-species pilot phase) with several centers set up to collect samples. In addition to these sampling centers, we would like to obtain further samples from across the world. We intend to make sure all samples are taken and transported with the full capture, licensing, and legal permits required from the appropriate authorities, in compliance with the permits of endangered and non-endangered species. Additionally, we will obtain prior informed consent for accessing genetic resources and sharing the benefits arising from this project (following the obligations of the Nagoya protocol). To make sure we have enough information for further analysis and to maximize the value of these genome data, we propose a sampling standard for the project. With associated metadata designed to include as much information as possible (including the source and geo-location), we will be stressing the importance of collecting images of each specimen, and of adequate storage conditions (frozen or voucher specimen). For sequencing, we propose to use both second- and third-generation sequencing technologies to generate high-quality genome assemblies. Based on our pilot study, and considering the feasibility of obtaining the required amount of high molecular DNA, for the majority of the species we have chosen a strategy combining stLFR data, low-depth Nanopore data, and Hi-C data (Strategy II in Fig. 2). For more complex genomes, we will generate high-depth Nanopore sequence data to ensure that good assemblies can be achieved (Strategy III, stLFR data + high-depth Nanopore data + Hi-C data; Fig. 2). For key species (to be determined by the working groups; see below), we will employ a Pacific Biosciences circular consensus sequencing, long, high-fidelity approach, allowing the generation of highly accurate long reads. For the large-scale sequencing of 6,000 species in Phase III, we propose to employ stLFR alone (Strategy I in Fig. 2). The genome assembly criteria will refer to the metric standard of reference genomes, for which we have grouping with the VGP (NCBI Bioproject PRJ489243).

### Fish10k data sharing

As per the Fort Lauderdale [8] and Toronto International Data Release Workshop guidelines [9], all sequencing data (including raw data, assemblies, and annotations) will be deposited in NCBI, as well as the GigaDB and China National Gene Bank repositories. The website of Fish10K (http://icg-ocean.genomics.cn/index.php/fish10kintroduction) will provide detailed information on the project status, as well as continuously updated information on the sequenced species. It also provides a portal for data downloads (particularly for assembled genomes).

### Organization of Fish10K consortium

Fish10K has been initiated by a core group of researchers that forms the steering committee of Fish10K (Fig. 3). The steering committee oversees the project and is responsible for fundraising, expanding the steering committee, organizing the scientific groups and sampling groups, coordinating sampling, identifying the sequencing centers where the sequencing will be done, assigning responsibilities (e.g., sequencing, assembly) to those centers, and creating analysis strategies. The steering committee is also responsible for the generation of genomic data. Various scientific groups will focus on technical and scientific questions related to this project. We wish to receive proposals from researchers who would like to take part in these scientific groups. We also invite researchers who are studying fish species which are rare or extinct to join Fish10K as members in the sampling group (with or without associated funding for sequencing). In addition to obtaining the genome sequences of their area of interest, joining the consortium provides immediate access to all genomes currently being assembled by Fish10K [10].

## Conclusion

Fish10K will generate an unprecedented, comprehensive data set of fish: the largest and most diverse vertebrate group. Our effort will allow us to complete the genomic tree for fish and, in concert with other projects, such as VGP and B10K, complete trees for vertebrates in general.

## Availability of supporting data and materials

The 10 fish genome assemblies in the pilot study have been deposited in the China National GeneBank (https://db.cngb.org/cnsa) with accession codes CNP0000597 and CNP0000691. The sequencing data of 10 fish are also deposited in NCBI with bioproject numbers PRJNA597275 and PRJNA558872. The individual genomes also all have individual DOIs in GigaDB, linked from a project page [10].

## Abbreviations

B10K: Bird 10,000 Genomes Project; BUSCO: benchmarking universal single-copy orthologs; Fish10K: 10,000 Fish Genomes Project; stLFR: single-tube long fragment reads technology; VGP: Vertebrate Genomes Project.

## Competing interests

Some of the authors are employees of BGI Group. The authors otherwise declare that they have no competing interests.

## Funding

This work was supported by the special funding of “Blue granary” scientific and technological innovation of China (2018YFD0900301-05).

## Authors' contributions

S.H., N.C., X.X., X.L., W.W., and G.F. conceived and designed the study. L.Y., M.Z. and S.L. performed sample collection and sequencing. Y.S., S.Z., and X.H. performed the assembly and annotation. X.L., Y.S., and G.F. wrote the manuscript. N.C. and all other authors revised and read the manuscript.

## Supplementary Material

giaa080_Supplemental_Tables

## Supplementary data

Supplementary Tables are available online.
