# Supplementary material for: Initial data release and announcement of the 10,000 Fish Genomes Project (Fish10K)
Source: Gigascience. 2020 Aug 18;9(8):giaa080. doi: 10.1093/gigascience/giaa080 (PMC7433795; doi:10.1093/gigascience/giaa080)
Supplement: giaa080_Supplemental_Tables [file giaa080_supplemental_tables.zip › Supplementary Tables.docx]

**Supplementary Tables**

**Supplementary Table 1. Assembly information of publicly available fish genomes.** Extant fishes are divided into three major classes, among which Actinopterygii (ray-finned fishes) account for more than half.

| species name | NCBI Taxonomy ID | Assembly level | Total sequence length (bp) | Number of scaffolds | Scaffold N50 (bp) | Number of contigs | Contig N50 (bp) | Accession  Number |
| --- | --- | --- | --- | --- | --- | --- | --- | --- |
| *Acanthochaenus luetkenii* | 473344 | Scaffold | 545,759,480 | 91,087 | 8,444 | 131,532 | 5,636 | GCA_900312575.1 |
| *Acanthochromis polyacanthus* | 80966 | Scaffold | 991,584,656 | 30,414 | 334,400 | 159,493 | 16,099 | GCF_002109545.1 |
| *Acipenser ruthenus* | 7906 | Scaffold | 1,732,545,901 | 215,913 | 219,750 | 446,905 | 24,377 | GCA_004119895.1 |
| *Ageneiosus marmoratus* | 2066578 | Scaffold | 1,030,000,983 | 16,063 | 223,139 | 169,048 | 7,741 | GCA_003347165.1 |
| *Amphilophus citrinellus* | 61819 | Scaffold | 844,902,565 | 6,637 | 1,216,136 | 67,543 | 23,456 | GCA_000751415.1 |
| *Amphiprion ocellaris* | 80972 | Scaffold | 880,720,895 | 6,405 | 401,715 | 7,803 | 324,210 | GCF_002776465.1 |
| *Amphiprion percula* | 161767 | Chromosome | 908,955,932 | 366 | 38,416,550 | 1,048 | 3,123,421 | GCA_003047355.2 |
| *Anabarilius grahami* | 495550 | Scaffold | 991,887,266 | 80,398 | 4,459,447 | 131,192 | 36,058 | GCA_003731715.1 |
| *Anarrhichthys ocellatus* | 433405 | Scaffold | 612,774,492 | 10,816 | 5,717,598 | 33,924 | 41,757 | GCA_004355925.1 |
| *Anguilla anguilla* | 7936 | Scaffold | 1,018,701,900 | 501,148 | 59,657 | 865,467 | 2,544 | GCA_000695075.1 |
| *Anguilla japonica* | 7937 | Scaffold | 966,917,315 | 83,292 | 36,264,158 | 276,102 | 11,014 | GCA_003597225.1 |
| *Anguilla rostrata* | 7938 | Scaffold | 1,413,032,609 | 79,209 | 86,641 | 307,316 | 7,355 | GCA_001606085.1 |
| *Anoplogaster cornuta* | 88656 | Scaffold | 404,844,788 | 108,625 | 4,562 | 128,159 | 3,719 | GCA_900683385.1 |
| *Anoplopoma fimbria* | 229290 | Contig | 699,326,415 |  |  | 208,506 | 5,156 | GCA_000499045.1 |
| *Antennarius striatus* | 241820 | Scaffold | 441,856,641 | 70,398 | 9,774 | 103,068 | 6,086 | GCA_900303275.1 |
| *Aphyosemion australe* | 52653 | Scaffold | 868,348,903 | 12,236 | 1,435,212 | 30,782 | 119,465 | GCA_006937985.1 |
| *Arapaima gigas* | 113544 | Scaffold | 667,351,951 | 60,052 | 285,171 | 70,752 | 64,954 | GCA_007844225.1 |
| *Archocentrus centrarchus* | 63155 | Chromosome | 932,930,362 | 188 | 35,590,001 | 926 | 2,146,538 | GCF_007364275.1 |
| *Arctogadus glacialis* | 185735 | Scaffold | 428,791,846 | 139,389 | 3,702 | 151,920 | 3,282 | GCA_900303235.1 |
| *Astatotilapia burtoni* | 8153 | Scaffold | 831,411,547 | 8,001 | 1,194,190 | 69,074 | 21,886 | GCF_000239415.1 |
| *Astatotilapia calliptera* | 8154 | Chromosome | 880,445,564 | 249 | 38,669,361 | 739 | 4,438,245 | GCF_900246225.1 |
| *Astyanax mexicanus* | 7994 | Chromosome | 1,335,239,194 | 2,415 | 35,377,769 | 3,030 | 1,767,240 | GCF_000372685.2 |
| *Austrofundulus limnaeus* | 52670 | Scaffold | 866,963,281 | 29,785 | 1,098,383 | 168,369 | 8,097 | GCF_001266775.1 |
| *Bathygadus melanobranchus* | 630650 | Scaffold | 431,202,967 | 92,290 | 6,483 | 112,630 | 4,956 | GCA_900302375.1 |
| *Benthosema glaciale* | 125796 | Scaffold | 676,314,385 | 143,923 | 6,111 | 188,319 | 4,393 | GCA_900323375.1 |
| *Beryx splendens* | 88663 | Scaffold | 533,267,752 | 117,400 | 5,987 | 151,933 | 4,286 | GCA_900312565.1 |
| *Betta splendens* | 158456 | Chromosome | 441,388,503 | 70 | 20,129,463 | 398 | 2,497,747 | GCF_900634795.2 |
| *Boleophthalmus pectinirostris* | 150288 | Scaffold | 955,752,150 | 16,620 | 2,375,582 | 108,947 | 20,437 | GCF_000788275.1 |
| *Boreogadus saida* | 44932 | Scaffold | 412,070,465 | 137,701 | 3,572 | 147,911 | 3,221 | GCA_900302515.1 |
| *Borostomias antarcticus* | 473354 | Scaffold | 430,362,827 | 104,734 | 5,368 | 133,045 | 3,928 | GCA_900323325.1 |
| *Bregmaceros cantori* | 630652 | Scaffold | 1,144,104,325 | 258,566 | 5,922 | 319,170 | 4,452 | GCA_900302395.1 |
| *Brosme brosme* | 81638 | Scaffold | 412,731,310 | 114,910 | 4,650 | 136,096 | 3,682 | GCA_900302425.1 |
| *Brotula barbata* | 432164 | Scaffold | 485,061,200 | 29,854 | 45,752 | 59,402 | 17,578 | GCA_900303265.1 |
| *Callopanchax toddi* | 60409 | Scaffold | 853,385,936 | 9,724 | 1,656,350 | 45,972 | 52,012 | GCA_006937965.1 |
| *Carapus acus* | 1491482 | Scaffold | 387,834,307 | 46,699 | 16,922 | 70,747 | 9,554 | GCA_900312935.1 |
| *Carassius auratus* | 7957 | Chromosome | 1,820,635,050 | 6,216 | 22,763,433 | 8,463 | 821,153 | GCF_003368295.1 |
| *Coryphaenoides rupestris* | 163118 | Scaffold | 829,208,733 | 47,680 | 159,738 | 82,633 | 20,848 | GCA_002895965.1 |
| *Cottoperca gobio* | 56716 | Chromosome | 609,391,784 | 322 | 25,156,145 | 766 | 6,330,900 | GCF_900634415.1 |
| *Cottus rhenanus* | 446433 | Scaffold | 563,609,416 | 164,693 | 7,249 | 490,620 | 2,129 | GCA_001455555.1 |
| *Cynoglossus semilaevis* | 244447 | Chromosome | 470,199,494 | 31,181 | 509,861 | 62,912 | 27,008 | GCF_000523025.1 |
| *Cyprinodon variegatus* | 28743 | Scaffold | 1,035,184,475 | 9,259 | 835,301 | 110,959 | 20,803 | GCF_000732505.1 |
| *Cyprinus carpio* | 7962 | Chromosome | 1,713,658,011 | 9,378 | 7,828,959 | 53,088 | 75,080 | GCF_000951615.1 |
| *Cyttopsis rosea* | 1176755 | Scaffold | 546,506,150 | 111,974 | 7,082 | 150,231 | 4,843 | GCA_900302355.1 |
| *Danio rerio* | 7955 | Chromosome | 1,373,454,788 | 1,917 | 7,379,053 | 19,725 | 1,422,317 | GCF_000002035.6 |
| *Danionella dracula* | 623740 | Scaffold | 665,208,374 | 996 | 10,287,669 | 1,611 | 2,300,191 | GCA_900490495.1 |
| *Danionella translucida* | 623744 | Scaffold | 735,303,417 | 27,639 | 340,819 | 36,005 | 133,131 | GCA_007224835.1 |
| *Denticeps clupeoides* | 299321 | Chromosome | 567,401,054 | 460 | 22,793,177 | 924 | 3,059,612 | GCF_900700375.1 |
| *Dicentrarchus labrax* | 13489 | Scaffold | 675,917,103 | 25 | 26,439,989 | 37,781 | 54,134 | GCA_000689215.1 |
| *Diretmoides pauciradiatus* | 1415272 | Scaffold | 672,603,014 | 147,686 | 6,032 | 204,934 | 4,028 | GCA_900660315.1 |
| *Diretmus argenteus* | 88682 | Scaffold | 302,363,458 | 107,968 | 3,367 | 162,384 | 2,172 | GCA_900660295.1 |
| *Echeneis naucrates* | 173247 | Chromosome | 544,229,245 | 38 | 23,287,306 | 178 | 12,371,513 | GCF_900963305.1 |
| *Electrophorus electricus* | 8005 | Scaffold | 551,880,868 | 8,786 | 613,956 | 47,652 | 37,141 | GCF_003665695.1 |
| *Epinephelus lanceolatus* | 310571 | Chromosome | 1,087,399,367 | 4,200 | 46,227,939 | 22,225 | 159,800 | GCA_005281545.1 |
| *Erpetoichthys calabaricus* | 27687 | Chromosome | 3,811,038,701 | 1,885 | 199,226,436 | 7,498 | 1,143,051 | GCF_900747795.1 |
| *Esox lucius* | 8010 | Chromosome | 940,906,975 | 811 | 37,550,661 | 1,395 | 3,396,779 | GCF_004634155.1 |
| *Fundulus heteroclitus* | 8078 | Scaffold | 1,021,898,560 | 10,180 | 1,252,252 | 120,723 | 16,688 | GCF_000826765.1 |
| *Gadiculus argenteus* | 185737 | Scaffold | 396,767,394 | 123,363 | 3,951 | 137,724 | 3,379 | GCA_900302595.1 |
| *Gadus chalcogrammus* | 1042646 | Scaffold | 448,868,398 | 130,159 | 4,335 | 149,286 | 3,603 | GCA_900302575.1 |
| *Gambusia affinis* | 33528 | Scaffold | 598,663,367 | 2,943 | 6,651,460 | 73,682 | 17,511 | GCA_003097735.1 |
| *Gasterosteus aculeatus* | 69293 | Scaffold | 467,452,432 | 10,242 | 3,715,221 | 32,646 | 38,090 | GCA_006229165.1 |
| *Gephyroberyx darwinii* | 334984 | Scaffold | 535,045,983 | 55,277 | 18,145 | 102,041 | 8,514 | GCA_900660455.1 |
| *Gouania willdenowi* | 441366 | Chromosome | 937,150,793 | 441 | 38,978,045 | 1,594 | 1,838,341 | GCF_900634775.1 |
| *Guentherus altivela* | 1263181 | Scaffold | 539,598,795 | 189,411 | 3,201 | 205,243 | 2,928 | GCA_900312595.1 |
| *Haplochromis nyererei* | 303518 | Scaffold | 830,133,247 | 7,236 | 2,525,540 | 68,053 | 22,622 | GCF_000239375.1 |
| *Hippocampus comes* | 109280 | Scaffold | 493,775,940 | 37,377 | 2,034,572 | 60,478 | 39,546 | GCF_001891065.1 |
| *Holocentrus rufus* | 722565 | Scaffold | 649,757,301 | 58,113 | 21,389 | 113,697 | 9,243 | GCA_900302615.1 |
| *Hoplostethus atlanticus* | 96778 | Scaffold | 520,173,038 | 58,279 | 15,562 | 103,281 | 7,812 | GCA_900660355.1 |
| *Hucho hucho* | 62062 | Scaffold | 2,487,549,814 | 71,639 | 287,338 | 221,746 | 37,639 | GCA_003317085.1 |
| *Hypophthalmichthys molitrix* | 13095 | Scaffold | 1,104,676,189 | 107,095 | 314,181 | 1,528,765 | 2,130 | GCA_004764525.1 |
| *Hypophthalmichthys nobilis* | 7965 | Scaffold | 1,012,063,666 | 121,326 | 83,012 | 742,098 | 4,840 | GCA_004193235.1 |
| *Hypoplectrus puella* | 146810 | Scaffold | 612,290,098 | 14,375 | 24,210,077 | 64,238 | 22,581 | GCA_900610375.1 |
| *Ictalurus punctatus* | 7998 | Chromosome | 1,002,389,428 | 3,163 | 26,676,597 | 5,816 | 2,695,784 | GCA_004006655.2 |
| *Kryptolebias hermaphroditus* | 1747188 | Chromosome | 683,986,837 | 5,211 | 27,459,464 | 32,872 | 46,411 | GCA_007896545.1 |
| *Kryptolebias marmoratus* | 37003 | Chromosome | 1,002,389,428 | 3,163 | 26,676,597 | 5,816 | 2,695,784 | GCA_004006655.2 |
| *Labeo rohita* | 84645 | Scaffold | 1,484,730,970 | 13,623 | 1,959,535 | 42,076 | 522,833 | GCA_004120215.1 |
| *Labeotropheus fuelleborni* | 57307 | Scaffold | 70,858,381 | 58,245 | 1,204 | 81,167 | 1,070 | GCA_000150875.1 |
| *Labrus bergylta* | 56723 | Scaffold | 805,480,521 | 13,466 | 794,648 | 13,723 | 703,847 | GCF_900080235.1 |
| *Laemonema laureysi* | 1784819 | Scaffold | 306,494,646 | 86,525 | 4,715 | 108,340 | 3,431 | GCA_900303225.1 |
| *Lampris guttatus* | 81370 | Scaffold | 849,277,706 | 208,230 | 5,222 | 253,854 | 4,051 | GCA_900302545.1 |
| *Lamprogrammus exutus* | 1592065 | Scaffold | 492,850,272 | 120,937 | 5,470 | 145,536 | 4,213 | GCA_900312555.1 |
| *Larimichthys crocea* | 215358 | Chromosome | 657,939,657 | 9,998 | 27,037,660 | 16,979 | 277,487 | GCF_000972845.2 |
| *Lates calcarifer* | 8187 | Scaffold | 668,481,366 | 3,808 | 1,191,366 | 3,918 | 1,066,117 | GCF_001640805.1 |
| *Lepisosteus oculatus* | 7918 | Chromosome | 945,878,036 | 2,106 | 6,928,108 | 45,200 | 68,337 | GCF_000242695.1 |
| *Lesueurigobius sanzi* | 1365564 | Scaffold | 810,626,388 | 130,360 | 11,480 | 186,717 | 6,729 | GCA_900303255.1 |
| *Leuciscus waleckii* | 155063 | Scaffold | 752,538,629 | 4,888 | 21,959,719 | 38,277 | 38,877 | GCA_900092035.1 |
| *Liparis tanakae* | 230148 | Scaffold | 498,979,456 | 27,878 | 375,216 | 97,972 | 9,903 | GCA_006348945.1 |
| *Lota lota* | 69944 | Scaffold | 397,499,185 | 106,616 | 4,892 | 128,281 | 3,803 | GCA_900302385.1 |
| *Maccullochella peelii* | 135761 | Scaffold | 633,241,041 | 18,198 | 109,974 | 31,008 | 70,439 | GCA_002120245.1 |
| *Macquaria australasica* | 135765 | Scaffold | 675,976,139 | 2,962 | 845,515 | 3,369 | 678,975 | GCA_005408345.1 |
| *Macrourus berglax* | 473319 | Scaffold | 399,875,629 | 118,318 | 4,291 | 142,500 | 3,353 | GCA_900302365.1 |
| *Malacocephalus occidentalis* | 630739 | Scaffold | 350,339,566 | 95,829 | 4,932 | 116,459 | 3,697 | GCA_900312585.1 |
| *Mastacembelus armatus* | 205130 | Chromosome | 591,935,101 | 122 | 25,090,313 | 358 | 8,014,513 | GCA_900324485.2 |
| *Maylandia zebra* | 106582 | Chromosome | 957,485,262 | 1,690 | 32,660,920 | 2,331 | 1,407,748 | GCF_000238955.4 |
| *Mchenga conophoros* | 35575 | Scaffold | 73,425,564 | 61,923 | 1,329 | 85,821 | 1,066 | GCA_000150855.1 |
| *Melanochromis auratus* | 27751 | Scaffold | 68,238,634 | 63,297 | 1,063 | 86,145 | 977 | GCA_000150895.1 |
| *Melanogrammus aeglefinus* | 8056 | Scaffold | 652,790,733 | 8,420 | 209,126 | 15,188 | 77,605 | GCA_900291075.1 |
| *Melanonus zugmayeri* | 181410 | Scaffold | 432,902,915 | 82,409 | 7,633 | 123,515 | 4,562 | GCA_900302345.1 |
| *Merlangius merlangus* | 8058 | Scaffold | 423,942,190 | 122,642 | 4,444 | 144,924 | 3,538 | GCA_900323355.1 |
| *Merluccius capensis* | 89947 | Scaffold | 414,317,329 | 110,925 | 4,774 | 131,629 | 3,792 | GCA_900312945.1 |
| *Merluccius merluccius* | 8063 | Scaffold | 401,034,705 | 102,914 | 5,117 | 133,682 | 3,670 | GCA_900312545.1 |
| *Merluccius polli* | 89951 | Scaffold | 401,149,128 | 113,894 | 4,482 | 138,586 | 3,471 | GCA_900312625.1 |
| *Micropterus floridanus* | 225391 | Contig | 1,001,521,525 |  |  | 249,768 | 10,978 | GCA_002592385.1 |
| *Miichthys miiuy* | 240162 | Scaffold | 619,300,777 | 6,294 | 1,145,539 | 20,386 | 81,271 | GCA_001593715.1 |
| *Mola mola* | 94237 | Scaffold | 639,451,992 | 5,552 | 8,766,736 | 51,826 | 23,239 | GCA_001698575.1 |
| *Molva molva* | 163112 | Scaffold | 437,480,619 | 111,875 | 5,266 | 133,189 | 4,136 | GCA_900323295.1 |
| *Monocentris japonica* | 181435 | Scaffold | 556,023,515 | 52,108 | 18,672 | 109,034 | 8,046 | GCA_900323365.1 |
| *Mora moro* | 248765 | Scaffold | 344,961,111 | 100,621 | 4,433 | 125,652 | 3,267 | GCA_900303205.1 |
| *Morone chrysops* | 46259 | Scaffold | 620,984,155 | 84,096 | 51,932 | 111,517 | 20,318 | GCA_003610055.1 |
| *Morone saxatilis* | 34816 | Scaffold | 598,109,547 | 629 | 25,942,274 | 70,506 | 17,211 | GCA_004916995.1 |
| *Muraenolepis marmorata* | 487677 | Scaffold | 416,390,766 | 138,928 | 3,555 | 152,594 | 3,126 | GCA_900302325.1 |
| *Myoxocephalus scorpius* | 8097 | Scaffold | 520,316,443 | 85,863 | 9,473 | 127,441 | 5,716 | GCA_900312955.1 |
| *Myripristis jacobus* | 371672 | Scaffold | 720,396,841 | 64,974 | 21,306 | 121,390 | 9,816 | GCA_900302555.1 |
| *Myripristis murdjan* | 586833 | Chromosome | 835,254,674 | 87 | 34,950,760 | 340 | 14,475,636 | GCF_902150065.1 |
| *Neogobius melanostomus* | 47308 | Contig | 1,003,738,541 |  |  | 1,364 | 2,817,412 | GCA_007210695.1 |
| *Neolamprologus brichardi* | 32507 | Scaffold | 847,910,432 | 9,099 | 4,430,025 | 118,197 | 13,047 | GCF_000239395.1 |
| *Nibea albiflora* | 240163 | Scaffold | 574,466,150 | 11,977 | 2,154,052 | 34,769 | 55,145 | GCA_900327885.1 |
| *Nothobranchius furzeri* | 105023 | Chromosome | 1,242,518,059 | 6,013 | 15,858,201 | 74,941 | 19,950 | GCF_001465895.1 |
| *Nothobranchius kuhntae* | 321403 | Scaffold | 1,122,656,415 | 34,756 | 1,178,460 | 101,919 | 22,234 | GCA_006942095.1 |
| *Notothenia coriiceps* | 8208 | Scaffold | 636,613,682 | 38,657 | 217,655 | 72,571 | 17,492 | GCF_000735185.1 |
| *Oncorhynchus kisutch* | 8019 | Chromosome | 2,369,932,239 | 22,813 | 1,266,128 | 97,074 | 58,118 | GCF_002021735.1 |
| *Oncorhynchus mykiss* | 8022 | Chromosome | 2,178,999,613 | 139,800 | 1,670,138 | 559,855 | 13,827 | GCF_002163495.1 |
| *Oncorhynchus nerka* | 8023 | Chromosome | 1,927,141,915 | 38,027 | 1,058,586 | 57,813 | 329,583 | GCF_006149115.1 |
| *Oncorhynchus tshawytscha* | 74940 | Chromosome | 2,425,713,975 | 15,946 | 1,728,323 | 69,485 | 133,169 | GCF_002872995.1 |
| *Ophiodon elongatus* | 225387 | Scaffold | 635,567,917 | 18,379 | 5,092,707 | 52,084 | 31,240 | GCA_004358465.1 |
| *Oplegnathus fasciatus* | 163134 | Scaffold | 766,301,214 | 4,149 | 1,126,915 | 66,839 | 29,992 | GCA_003416845.1 |
| *Opsanus beta* | 95145 | Scaffold | 1,028,783,780 | 345,629 | 3,335 | 371,656 | 3,062 | GCA_900660325.1 |
| *Oreochromis aureus* | 47969 | Scaffold | 918,937,175 | 12,951 | 1,102,239 | 61,878 | 60,340 | GCA_005870065.1 |
| *Oreochromis niloticus* | 8128 | Chromosome | 1,005,681,550 | 2,460 | 38,839,487 | 3,010 | 2,923,640 | GCF_001858045.2 |
| *Oreochromis spilurus* | 64544 | Contig | 764,974,731 |  |  | 221,829 | 11,851 | GCA_008269305.1 |
| *Oryzias javanicus* | 123683 | Chromosome | 809,679,899 | 254 | 35,390,520 | 751 | 3,558,013 | GCA_003999625.1 |
| *Oryzias latipes* | 8090 | Chromosome | 734,057,086 | 25 | 31,218,526 | 516 | 2,530,934 | GCF_002234675.1 |
| *Oryzias melastigma* | 30732 | Scaffold | 779,469,774 | 8,603 | 23,737,187 | 56,275 | 30,057 | GCF_002922805.1 |
| *Osmerus eperlanus* | 29151 | Scaffold | 342,758,722 | 73,274 | 6,820 | 99,348 | 4,524 | GCA_900302275.1 |
| *Oxygymnocypris stewartii* | 361644 | Scaffold | 1,849,224,471 | 26,281 | 257,093 | 26,283 | 257,093 | GCA_003573665.1 |
| *Pachypanchax playfairii* | 52664 | Scaffold | 669,774,067 | 4,488 | 3,173,794 | 19,608 | 76,641 | GCA_006937955.1 |
| *Pagrus major* | 143350 | Scaffold | 875,465,402 | 886,260 | 4,644 | 1,164,424 | 2,822 | GCA_002897255.1 |
| *Pampus argenteus* | 206143 | Scaffold | 350,448,509 | 298,139 | 1,586 | 532,813 | 1,001 | GCA_000697985.1 |
| *Pangasianodon hypophthalmus* | 310915 | Scaffold | 715,760,110 | 567 | 14,288,580 | 23,339 | 62,522 | GCF_003671635.1 |
| *Parablennius parvicornis* | 171872 | Scaffold | 599,249,148 | 66,539 | 16,796 | 129,811 | 7,343 | GCA_900302745.1 |
| *Paralichthys olivaceus* | 8255 | Chromosome | 545,775,252 | 7,202 | 3,817,360 | 38,614 | 30,544 | GCA_001904815.2 |
| *Parambassis ranga* | 210632 | Chromosome | 551,012,959 | 156 | 22,993,012 | 1,677 | 5,080,925 | GCF_900634625.1 |
| *Paramormyrops kingsleyae* | 1676925 | Scaffold | 799,421,083 | 4,667 | 1,731,158 | 47,999 | 37,656 | GCF_002872115.1 |
| *Parasudis fraserbrunneri* | 1784818 | Scaffold | 707,987,062 | 156,389 | 6,391 | 213,462 | 4,177 | GCA_900302295.1 |
| *Perca flavescens* | 8167 | Chromosome | 877,456,336 | 268 | 37,412,490 | 1,097 | 4,268,950 | GCF_004354835.1 |
| *Perca fluviatilis* | 8168 | Scaffold | 958,225,486 | 31,105 | 6,260,519 | 100,821 | 18,196 | GCA_003412525.1 |
| *Percopsis transmontana* | 143327 | Scaffold | 458,089,168 | 53,197 | 15,180 | 88,141 | 8,161 | GCA_900302285.1 |
| *Periophthalmodon schlosseri* | 1365757 | Scaffold | 679,761,122 | 46,662 | 39,308 | 85,749 | 16,946 | GCA_000787095.1 |
| *Periophthalmus magnuspinnatus* | 409849 | Scaffold | 701,696,780 | 26,060 | 296,161 | 76,770 | 28,254 | GCA_000787105.1 |
| *Phycis blennoides* | 163115 | Scaffold | 416,766,999 | 62,684 | 10,640 | 132,164 | 4,532 | GCA_900302315.1 |
| *Phycis phycis* | 349666 | Scaffold | 346,335,180 | 100,771 | 4,502 | 120,694 | 3,458 | GCA_900302335.1 |
| *Pimephales promelas* | 90988 | Scaffold | 1,219,326,373 | 73,057 | 60,380 | 215,176 | 7,468 | GCA_000700825.1 |
| *Planiliza haematocheila* | 370040 | Contig | 747,342,729 |  |  | 1,453 | 3,973,280 | GCA_005024645.1 |
| *Poecilia formosa* | 48698 | Scaffold | 748,923,461 | 3,985 | 1,574,226 | 31,058 | 57,472 | GCF_000485575.1 |
| *Poecilia latipinna* | 48699 | Scaffold | 815,144,743 | 17,988 | 279,200 | 54,625 | 33,278 | GCF_001443285.1 |
| *Poecilia mexicana* | 48701 | Scaffold | 801,711,499 | 18,105 | 275,316 | 50,601 | 39,840 | GCF_001443325.1 |
| *Poecilia reticulata* | 8081 | Chromosome | 731,622,281 | 3,029 | 5,270,359 | 40,144 | 41,908 | GCF_000633615.1 |
| *Pollachius virens* | 8060 | Scaffold | 394,927,939 | 116,705 | 4,344 | 137,332 | 3,457 | GCA_900312635.1 |
| *Polymixia japonica* | 81385 | Scaffold | 554,895,936 | 92,198 | 9,571 | 134,725 | 5,803 | GCA_900302305.1 |
| *Poropuntius huangchuchieni* | 357532 | Scaffold | 760,177,161 | 625,277 | 2,931 | 821,804 | 2,273 | GCA_004124795.1 |
| *Pseudochromis fuscus* | 280673 | Scaffold | 657,041,210 | 52,042 | 24,689 | 91,797 | 12,029 | GCA_900323345.1 |
| *Pseudopleuronectes yokohamae* | 245875 | Contig | 547,831,023 |  |  | 525,502 | 1,994 | GCA_000787555.1 |
| *Pungitius pungitius* | 134920 | Scaffold | 441,089,565 | 7,847 | 302,682 | 49,352 | 14,136 | GCA_003399555.1 |
| *Pygocentrus nattereri* | 42514 | Scaffold | 1,285,352,492 | 283,518 | 1,440,044 | 325,620 | 57,732 | GCF_001682695.1 |
| *Regalecus glesne* | 81389 | Scaffold | 656,003,707 | 105,196 | 9,773 | 141,447 | 6,781 | GCA_900302585.1 |
| *Reinhardtius hippoglossoides* | 111784 | Scaffold | 677,540,803 | 4,453 | 17,640,195 | 5,488 | 775,256 | GCA_006182925.2 |
| *Rhamphochromis esox* | 163638 | Scaffold | 71,295,074 | 55,751 | 1,324 | 78,130 | 1,126 | GCA_000150935.1 |
| *Rondeletia loricata* | 88713 | Scaffold | 568,597,941 | 103,827 | 7,469 | 140,955 | 5,112 | GCA_900302605.1 |
| *Salarias fasciatus* | 181472 | Chromosome | 797,507,141 | 203 | 32,729,575 | 805 | 2,597,836 | GCF_902148845.1 |
| *Salmo salar* | 8030 | Chromosome | 2,966,890,203 | 241,573 | 1,366,254 | 368,060 | 57,618 | GCA_000233375.4 |
| *Salmo trutta* | 8032 | Chromosome | 2,371,880,186 | 1,441 | 52,209,666 | 5,378 | 1,703,178 | GCF_901001165.1 |
| *Salvelinus alpinus* | 8036 | Chromosome | 2,169,553,147 | 16,702 | 1,018,695 | 97,014 | 55,619 | GCF_002910315.2 |
| *Sander lucioperca* | 283035 | Scaffold | 900,461,225 | 1,312 | 4,929,547 | 1,347 | 4,695,595 | GCA_008315115.1 |
| *Sardina pilchardus* | 27697 | Scaffold | 949,617,276 | 117,259 | 96,617 | 194,510 | 9,398 | GCA_900499035.1 |
| *Scartelaos histophorus* | 166764 | Scaffold | 695,008,792 | 156,044 | 15,105 | 209,353 | 8,806 | GCA_000787155.1 |
| *Scleropages formosus* | 113540 | Chromosome | 784,563,014 | 72 | 31,084,684 | 217 | 9,102,216 | GCF_900964775.1 |
| *Scophthalmus maximus* | 52904 | Chromosome | 524,979,463 | 22 | 24,811,384 | 21,326 | 54,836 | GCA_003186165.1 |
| *Sebastes aleutianus* | 214485 | Scaffold | 899,650,391 | 10,489 | 340,062 | 110,635 | 10,838 | GCA_001910805.2 |
| *Sebastes koreanus* | 290523 | Contig | 725,092,264 |  |  | 147,157 | 16,662 | GCA_004335335.1 |
| *Sebastes minor* | 214483 | Scaffold | 681,652,711 | 166,448 | 7,676 | 812,852 | 1,901 | GCA_001910765.2 |
| *Sebastes nigrocinctus* | 72089 | Scaffold | 746,044,620 | 15,872 | 116,274 | 89,356 | 13,471 | GCA_000475235.3 |
| *Sebastes norvegicus* | 394699 | Scaffold | 717,740,616 | 75,627 | 16,564 | 117,709 | 9,467 | GCA_900302655.1 |
| *Sebastes nudus* | 1617787 | Contig | 724,045,237 |  |  | 180,312 | 11,290 | GCA_004335365.1 |
| *Sebastes rubrivinctus* | 72099 | Scaffold | 756,296,653 | 68,206 | 30,046 | 136,109 | 13,541 | GCA_000475215.1 |
| *Sebastes schlegelii* | 214486 | Contig | 728,476,695 |  |  | 146,105 | 14,246 | GCA_004335315.1 |
| *Sebastes steindachneri* | 201708 | Scaffold | 648,011,071 | 279,232 | 4,288 | 1,089,366 | 1,311 | GCA_001910785.2 |
| *Selene dorsalis* | 179366 | Scaffold | 528,779,420 | 36,113 | 32,464 | 85,660 | 11,209 | GCA_900303245.1 |
| *Seriola dumerili* | 41447 | Scaffold | 677,686,174 | 34,656 | 5,812,906 | 41,188 | 249,509 | GCF_002260705.1 |
| *Seriola lalandi* | 302047 | Scaffold | 766,364,468 | 7,606 | 411,616 | 63,278 | 37,711 | GCA_003054885.1 |
| *Seriola quinqueradiata* | 8161 | Scaffold | 639,269,536 | 384 | 5,610,255 | 1,312 | 872,227 | GCA_002217815.1 |
| *Seriola rivoliana* | 173321 | Scaffold | 666,141,578 | 1,343 | 9,509,606 | 3,939 | 740,108 | GCA_002994505.1 |
| *Simochromis diagramma* | 43689 | Scaffold | 848,827,444 | 823 | 8,960,300 | 1,764 | 2,231,376 | GCA_900408965.1 |
| *Sinocyclocheilus anshuiensis* | 1608454 | Scaffold | 1,632,718,266 | 85,682 | 1,284,143 | 254,423 | 17,271 | GCF_001515605.1 |
| *Sinocyclocheilus grahami* | 75366 | Scaffold | 1,750,287,761 | 31,277 | 1,156,368 | 168,074 | 29,353 | GCF_001515645.1 |
| *Sinocyclocheilus rhinocerous* | 307959 | Scaffold | 1,655,786,410 | 164,173 | 945,738 | 314,963 | 18,758 | GCF_001515625.1 |
| *Sparus aurata* | 8175 | Chromosome | 833,595,063 | 176 | 35,791,275 | 1,224 | 2,862,625 | GCF_900880675.1 |
| *Sphaeramia orbicularis* | 375764 | Chromosome | 1,342,662,642 | 340 | 57,165,184 | 2,183 | 2,360,121 | GCF_902148855.1 |
| *Spondyliosoma cantharus* | 50595 | Scaffold | 680,472,139 | 47,064 | 28,198 | 97,735 | 11,633 | GCA_900302685.1 |
| *Squalius pyrenaicus* | 263744 | Contig | 48,139,320 |  |  | 40,926 | 1,710 | GCA_001403095.1 |
| *Stegastes partitus* | 144197 | Scaffold | 800,491,834 | 5,818 | 411,659 | 42,060 | 43,010 | GCF_000690725.1 |
| *Stylephorus chordatus* | 409996 | Scaffold | 488,488,587 | 128,468 | 4,684 | 170,584 | 3,373 | GCA_900312615.1 |
| *Symphodus melops* | 171736 | Scaffold | 533,823,763 | 50,156 | 21,275 | 95,080 | 9,362 | GCA_900323315.1 |
| *Syngnathus acus* | 161584 | Chromosome | 324,331,233 | 87 | 14,974,571 | 130 | 11,959,915 | GCA_901709675.1 |
| *Tachysurus fulvidraco* | 1234273 | Scaffold | 713,810,725 | 663 | 3,653,474 | 2,402 | 980,445 | GCF_003724035.1 |
| *Takifugu bimaculatus* | 433685 | Chromosome | 371,675,663 | 22 | 16,786,025 | 1,055 | 1,398,332 | GCA_004026145.1 |
| *Takifugu flavidus* | 433684 | Chromosome | 366,286,831 | 867 | 15,676,631 | 1,111 | 4,357,567 | GCA_003711565.2 |
| *Takifugu rubripes* | 31033 | Chromosome | 384,126,662 | 128 | 16,705,553 | 530 | 3,136,617 | GCF_901000725.2 |
| *Tenualosa ilisha* | 373995 | Scaffold | 815,647,530 | 124,209 | 188,026 | 131,117 | 129,889 | GCA_003651195.1 |
| *Thalassoma bifasciatum* | 76338 | Scaffold | 1,095,910,316 | 379,332 | 155,821 | 397,893 | 122,955 | GCA_008086565.1 |
| *Thunnus albacares* | 8236 | Scaffold | 728,212,003 | 38,995 | 46,920 | 84,919 | 16,808 | GCA_900302625.1 |
| *Thunnus orientalis* | 8238 | Contig | 684,497,465 |  |  | 133,062 | 8,235 | GCA_000418415.1 |
| *Thunnus thynnus* | 8237 | Scaffold | 648,208,697 | 354,425 | 3,045 | 450,338 | 2,430 | GCA_003231725.1 |
| *Thymallus thymallus* | 36185 | Chromosome | 1,564,834,359 | 3,831 | 32,985,317 | 204,386 | 31,774 | GCA_004348285.1 |
| *Trachinotus ovatus* | 173339 | Scaffold | 648,062,395 | 138 | 29,494,812 | 749 | 1,846,793 | GCA_900607315.1 |
| *Trachyrincus murrayi* | 241836 | Scaffold | 452,416,606 | 40,927 | 19,998 | 114,476 | 6,231 | GCA_900323305.1 |
| *Trachyrincus scabrus* | 562814 | Scaffold | 369,861,760 | 80,958 | 6,379 | 119,164 | 3,900 | GCA_900303215.1 |
| *Triplophysa siluroides* | 422203 | Scaffold | 583,428,323 | 1,002 | 2,872,994 | 1,039 | 2,549,348 | GCA_006030095.1 |
| *Trisopterus minutus* | 80722 | Scaffold | 334,717,091 | 106,116 | 3,976 | 122,084 | 3,248 | GCA_900302415.1 |
| *Typhlichthys subterraneus* | 940470 | Scaffold | 555,559,596 | 84,841 | 9,654 | 106,331 | 7,314 | GCA_900302405.1 |
| *Xiphophorus couchianus* | 32473 | Chromosome | 688,541,509 | 68 | 30,550,352 | 297 | 15,315,838 | GCF_001444195.1 |
| *Xiphophorus hellerii* | 8084 | Chromosome | 733,126,988 | 85 | 26,465,357 | 561 | 7,119,835 | GCA_003331165.1 |
| *Zeus faber* | 64108 | Scaffold | 610,433,400 | 135,758 | 6,332 | 172,424 | 4,642 | GCA_900323335.1 |
| *Amblyraja radiata* | 386614 | Chromosome | 2,558,801,738 | 958 | 62,052,929 | 4,673 | 1,462,921 | GCA_010909765.1 |
| *Callorhinchus milii* | 7868 | Scaffold | 974,498,586 | 21,204 | 4,521,921 | 67,421 | 46,577 | GCF_000165045.1 |
| *Latimeria chalumnae* | 7897 | Scaffold | 2,736,328,441 | 37,861 | 331,424 | 475,424 | 11,394 | GCA_000325985.2 |
| *Rhincodon typus* | 259920 | Scaffold | 2,931,599,576 | 57,334 | 144,422 | 57,344 | 144,422 | GCF_001642345.1 |
| *Eptatretus burgeri* | 7764 | Scaffold | 2,608,383,542 | 10,846 | 2,692,996 | 341,068 | 7,991 | GCA_900186335.2 |
| *Pristis pectinata* | 685728 | Chromosome | 2,267,857,790 | 173 | 101,699,621 | 871 | 17,009,522 | GCA_009764475.1 |
| *Chiloscyllium punctatum* | 137246 | Scaffold | 3,375,698,300 | 280,241 | 1,963,924 | 461,670 | 21,276 | GCA_003427335.1 |
| *Chiloscyllium plagiosum* | 36176 | Chromosome | 3,776,550,609 | 601,049 | 72,140,027 | 756,729 | 37,239 | GCA_004010195.1 |
| *Scyliorhinus torazame* | 75743 | Scaffold | 4,470,980,620 | 458,049 | 282,424 | 1,040,580 | 7,146 | GCA_003427355.1 |
| *Protosalanx chinensis* | 240822 | Scaffold | 466,693,640 | 1,775 | 5,188,763 | 20,854 | 103,007 | GCA_010882115.1 |
| *Liparis tanakae* | 230148 | Scaffold | 498,979,456 | 27,878 | 375,216 | 97,972 | 9,903 | GCA_006348945.1 |
| *Poeciliopsis turrubarensis* | 188136 | Scaffold | 596,996,646 | 5,396 | 4,228,945 | 19,105 | 101,021 | GCA_010277015.1 |
| *Poeciliopsis retropinna* | 188133 | Scaffold | 621,858,691 | 78 | 21,601,530 | 156 | 15,063,430 | GCA_010277075.1 |
| *Gymnarchus niloticus* | 42648 | Scaffold | 653,962,364 | 54,167 | 1,179,548 | 63,532 | 54,271 | GCA_009936485.1 |
| *Larimichthys polyactis* | 334908 | Scaffold | 694,446,753 | 107,847 | 146,739 | 172,294 | 8,079 | GCA_010119295.1 |
| *Poeciliopsis occidentalis* | 68464 | Scaffold | 724,239,826 | 15,101 | 1,540,873 | 32,670 | 56,887 | GCA_010883035.1 |
| *Anguilla megastoma* | 86963 | Scaffold | 877,765,645 | 40,052 | 61,910 | 113,725 | 14,572 | GCA_901111305.1 |
| *Anguilla marmorata* | 7939 | Scaffold | 882,006,954 | 38,756 | 64,942 | 105,065 | 16,529 | GCA_901111315.1 |
| *Anguilla obscura* | 86964 | Scaffold | 882,390,062 | 43,284 | 54,849 | 128,070 | 12,681 | GCA_901111295.1 |
| *Culter alburnus* | 194366 | Scaffold | 1,017,706,352 | 5,625 | 3,669,388 | 22,738 | 121,415 | GCA_009869775.1 |
| *Lethenteron camtschaticum* | 980415 | Scaffold | 1,030,662,718 | 86,125 | 1,051,965 | 179,499 | 9,240 | GCA_000466285.1 |
| *Megalobrama amblycephala* | 75352 | Scaffold | 1,087,899,576 | 18,243 | 1,403,021 | 24,986 | 322,873 | GCA_009869865.1 |
| *Petromyzon marinus* | 7757 | Chromosome | 1,089,034,212 | 1,433 | 12,997,950 | 2,363 | 2,540,784 | GCA_010993605.1 |

**Supplementary Table 2. Statistics of the existing fish species.** Based on data from FishBase web site (accessed 10 September 2019) and “Fishes of the world” (5th ed.). Actinopterygii belong to Osteichthyes, whose number of species accounts for most all fishes.

| Type | Class | Order | Family | Species |
| --- | --- | --- | --- | --- |
| Chondrichthyes | Elasmobranchii | 12 | 51 | 1,190 |
|  | Holocephali | 1 | 3 | 56 |
| Osteichthyes | Actinopterygii | 67 | 475 | 32,869 |
| Total |  | 80 | 529 | 34,115 |
